# Association of adiposity indicators with cardiometabolic multimorbidity risk in hypertensive patients: a large cross-sectional study

**DOI:** 10.3389/fendo.2024.1302296

**Published:** 2024-03-21

**Authors:** Ting Dong, Weiquan Lin, Qin Zhou, Yunou Yang, Xiangyi Liu, Jiamin Chen, Hui Liu, Caixia Zhang

**Affiliations:** ^1^ Department of Epidemiology, School of Public Health, Sun Yat-sen University, Guangzhou, China; ^2^ Department of Basic Public Health, Guangzhou Center for Disease Control and Prevention, Guangzhou, China

**Keywords:** cardiometabolic multimorbidity, cardiometabolic index, lipid accumulation product, visceral adiposity index, Chinese visceral adiposity index, hypertension

## Abstract

**Background:**

Cardiometabolic multimorbidity (CMM) has emerged as a prominent public health concern. Hypertensive patients are prone to develop comorbidities. Moreover, the accumulation of visceral adipose tissue is the main cause for the development of cardiometabolic diseases. The cardiometabolic index (CMI), lipid accumulation product (LAP), visceral adiposity index (VAI), and Chinese visceral adiposity index (CVAI) not only assess adipose tissue mass but also reflect adipose tissue dysfunction. So far, no study has been reported to evaluate the association of CMI, LAP, VAI, and CVAI with CMM risk in hypertensive patients. Therefore, this study aimed to assess the association between these adiposity indicators and the risk of CMM among Chinese hypertensive patients.

**Methods:**

In this cross-sectional study, a total of 229,287 hypertensive patients aged 35 years and older were included from the National Basic Public Health Service Project. All participants underwent a face-to-face questionnaire survey, physical examination, and the collection of fasting venous blood samples. Multivariable logistic regression models were performed to estimate odds ratios (ORs) and 95% confidence intervals (CIs). Receiver operating characteristic curve was utilized to evaluate the identification ability for CMM.

**Results:**

After adjusting for confounders, each 1-standard deviation increase in CMI, LAP, VAI, and CVAI was associated with a 14%, 8%, 12%, and 54% increased risk of CMM, respectively. When comparing the highest quartile of these indicators with the lowest quartile, individuals in the highest quartile of CMM, LAP, VAI, and CVAI had a 1.39-fold (95% CI 1.30, 1.48), 1.28-fold (95% CI 1.19, 1.37), 1.37-fold (95% CI 1.29, 1.46), and 2.56-fold (95% CI 2.34, 2.79) increased risk of CMM after adjusting for potential confounders. Notably, a nonlinear association was observed for CMI, LAP, and VAI with the risk of CMM (all *P* nonlinearity < 0.001). CVAI exhibited the highest area under the receiver operating characteristic curve (AUC) among all the included adiposity indices in this analysis.

**Conclusion:**

This study indicated the significant positive association of CMI, LAP, VAI, and CVAI with the risk of CMM in hypertensive patients. Among these indicators, CVAI demonstrated the most robust performance in predicting CMM risk and may serve as a valuable tool for identifying CMM risk in Chinese hypertensive patients.

## 1 Introduction

Cardiometabolic multimorbidity (CMM), characterized by the simultaneous presence of at least two cardiometabolic conditions such as hypertension, diabetes, coronary heart disease, and stroke, represents a prevalent and severe form of multimorbidity (1, 2). The prevalence of CMM has been on a rapid rise, largely driven by the increasing proportion of aging individuals in the global population (3, 4). Studies conducted on a global scale, within the United Kingdom, and among Chinese populations have consistently demonstrated that CMM is associated with decreased life expectancy and an elevated risk of all-cause mortality (2, 5, 6). Given its high prevalence and adverse outcomes, CMM has emerged as a prominent concern in the field of public health. Furthermore, hypertension, which is the most prevalent chronic condition worldwide, is highly predisposed to the development of comorbidities (7). Approximately one quarter of hypertensive patients go on to develop CMM (8). Research has indicated that in hypertensive patients, the risk of all-cause mortality substantially rises, increasing from 7% to 30% when combined with diabetes and reaching 136% in the presence of cardiovascular disease (6). As a result, evaluating the risk of CMM in the hypertensive population holds paramount importance.

Mounting evidence suggests the accumulation of visceral adipose tissue as the primary cause for the development of cardiometabolic disorders (9). Nowadays, imaging techniques like magnetic resonance imaging and computed tomography offer quantitative assessments of visceral adipose tissue and fat distribution (10). However, these techniques are inappropriate for routine medical screenings due to their time-consuming nature, high costs, and potential radiation risks. Therefore, there is a demand for a simple and cost-effective predictor to identify hypertensive patients at high risk of CMM.

In recent years, there has been the development of several novel adiposity indices, namely the cardiometabolic index (CMI), lipid accumulation product (LAP), visceral adiposity index (VAI), and Chinese visceral adiposity index (CVAI). These indices not only measure adipose tissue mass but also provide insights into adipose tissue dysfunction (11–13). In 2015, Wakabayashi et al. proposed the concept of CMI, which incorporates waist-to-height ratio (WHtR), triglyceride (TG), and high-density lipoprotein cholesterol ratio (HDL-C) as a novel index to evaluate both the distribution and dysfunction of visceral adipose tissue (11, 14). LAP, based on a combination of waist circumference (WC) and TG levels, was proposed as a more accurate continuous index for describing lipid overaccumulation associated with central obesity and metabolic risk (13, 15). VAI, comprising WC, body mass index (BMI), TG, and HDL-C, allows for the measurement of abdominal fat distribution and has shown significant associations with cardiovascular and cerebrovascular events, as well as all factors of metabolic syndrome (12, 16). CVAI, a simple clinical index consisting of age, BMI, WC, TG, and HDL-C, has been considered as a surrogate biomarker for assessing visceral fat accumulation (17).

Previous studies have examined the association of CMI, LAP, VAI, and CVAI with the risk of individual cardiovascular disease in the general population, including conditions like hypertension, diabetes, and stroke (18–22). To the best of our knowledge, there has been no prior study that has specifically evaluated the association of CMI, LAP, VAI, and CVAI with CMM, particularly among individuals with hypertension. To address this research gap, the primary objective of this study was to evaluate how CMI, LAP, VAI and CVAI are associated with the risk of CMM in hypertensive populations. We hypothesize that higher levels of CMI, LAP, VAI, and CVAI are associated with a higher risk of CMM. The findings from our study are expected to provide valuable insights into the roles of CMI, LAP, VAI, and CVAI in CMM risk, potentially informing targeted strategies for the prevention of cardiometabolic diseases.

## 2 Methods

### 2.1 Study population and design

This study is based on the National Basic Public Health Service Project (NBPHS), which represents a core healthcare service provided by the government, free of charge, to all residents. Its primary objective is to cater to the primary health requirements of both urban and rural residents, with a specific focus on children, pregnant women, the elderly, and individuals with chronic health conditions (23).

**Figure 1:**
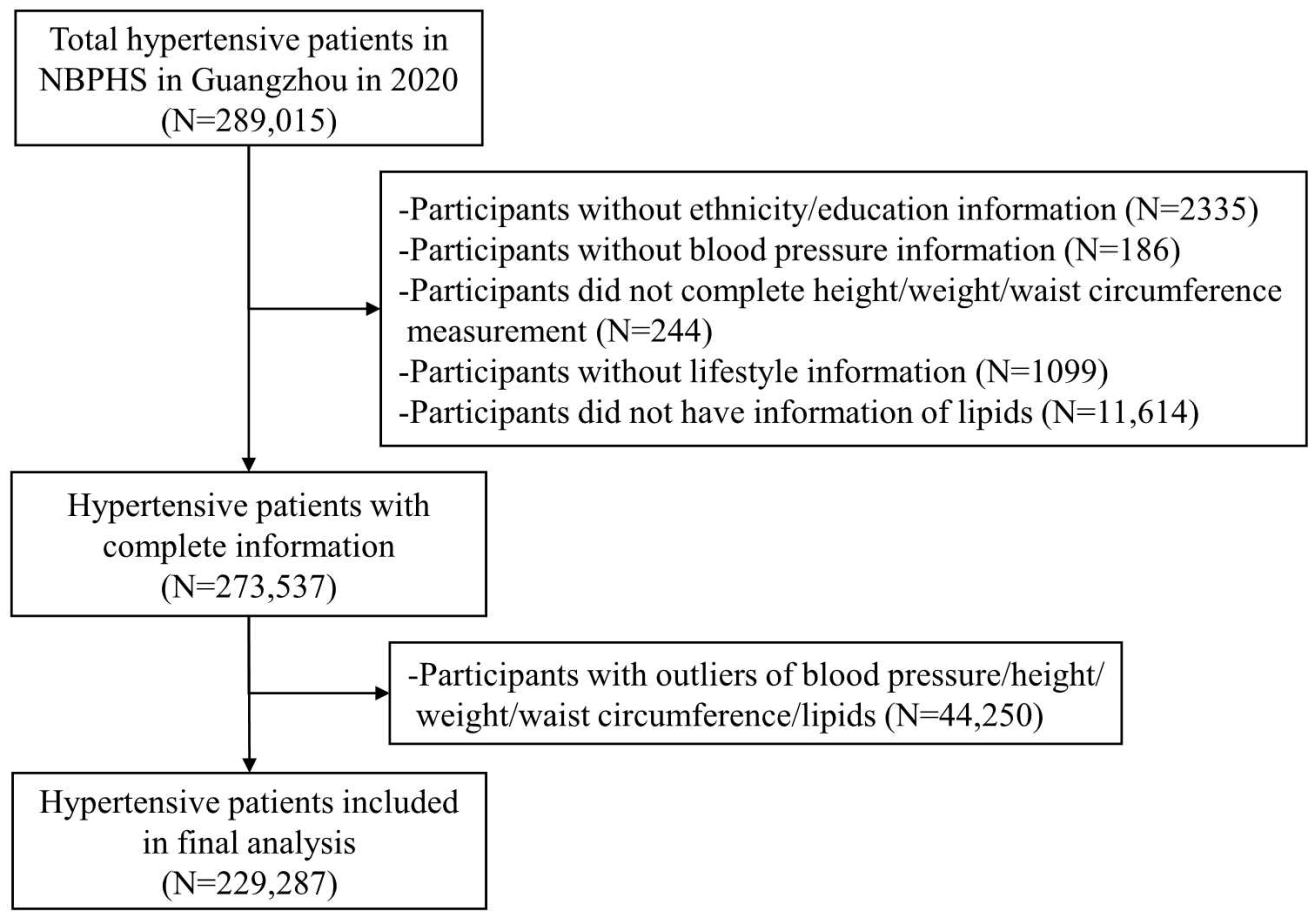
Study flow chart.

A total of 289,015 individuals who were recipients of the NBPHS in Guangzhou in the year 2020 were enrolled in this study. Inclusion criteria were as follows: individuals aged 35 years or older, Guangdong natives or residents with a minimum of six months’ residency in Guangzhou, individuals diagnosed with primary hypertension, and those who had undergone a physical examination. Hypertension was defined as having a resting blood pressure of ≥ 140/90 mmHg, current use of antihypertensive drugs, or self-reported hypertension (24). To assess this, participants received three separate blood pressure measurements on different days. A diagnosis of hypertension was confirmed if all three readings showed a blood pressure of ≥140/90 mmHg. Exclusions were made for participants who lacked information on ethnicity or education (n=2335); those without measurements of height, weight, WC, or blood pressure (n=430); individuals with missing data related to lifestyle and lipids levels (n=12,713); and those with outliers in blood pressure, height, weight, WC, and lipid measurements (n=44,250). Consequently, a total of 229,287 participants were included in the final analysis (**Figure 1**).

This study received approval from the Ethical Review Committee for School of Public Health, Sun Yat-sen University (approval number: 2023-007). The study was performed in accordance with the principles outlined in the Declaration of Helsinki.

### 2.2 Data collection and measurements

All participants underwent face-to-face interviews conducted by trained healthcare staff at community-level healthcare facilities using a standardized questionnaire. The collected information included socio-demographic characteristics (age, sex, ethnicity, educational level, and marital status) as well as lifestyle factors (dietary habits, physical activity, smoking habits, and alcohol drinking). Dietary habits were categorized as either healthy or unhealthy. Healthy dietary habits were characterized by a balanced intake of meat and vegetable, moderate consumption of salt, oil, and added sugar, while unhealthy dietary habits were identified as a preference for meat or a vegetarian diet, along with high consumption of salt, oil, or added sugar (25). Active physical activity was defined as engaging in at least 150 minutes per week of moderate activity or at least 75 minutes per week of vigorous activity (or a comparable combination of both) (26). Smoking status was divided into two groups: never smokers and smokers (comprising both former and current smokers). Similarly, drinking status was classified into never drinkers and drinkers (including both ever and current drinkers). Individuals were classified as drinkers if they consumed 10 grams or more of alcohol per day (27).

Standardized methods were used to measure the height, weight, WC, and blood pressure of the participants. BMI was calculated by dividing body weight in kilograms by the square of height in meters. WHtR was calculated as the ratio of WC to height in meters. systolic blood pressure (SBP) and diastolic blood pressure (DBP) were measured while participants were in a seated position, with readings taken from both arms. The mean value of the two measurements obtained from the left and right arms was considered as the individual’s blood pressure readings.

Fasting venous blood sample was collected, and the serum concentrations of total cholesterol (TC), TG, HDL-C, and low-density lipoprotein cholesterol (LDL-C) were measured using an automatic biochemical analyzer.

### 2.3 Definition of adiposity indicators

In our study, we evaluated three adiposity variables, namely CMI, LAP, VAI, and CVAI, through indirect calculations using relevant anthropometric parameters. TG/HDL-C was computed as the ratio of TG to HDL-C. CMI was derived as TG/HDL-C multiplied by WHtR (11). LAP was a sex-specific index calculated as follows: for males, LAP = [WC (cm)-65] × TG (mmol/L), and for females, LAP = [WC (cm)-58] × TG (mmol/L) (13). The formulas for VAI calculation were as follows: for males, VAI=WC (cm)/[39.68 + 1.88 × BMI (kg/m^2^)] × TG (mmol/L)/1.03 × 1.31/HDL-C (mmol/L); for females, VAI=WC (cm)/[(36.58 + 1.89 × BMI (kg/m^2^)] × TG (mmol/L)/0.81 × 1.52/HDL-C (mmol/L) (12). CVAI was computed using sex-specific formulas as follows: for males, CVAI= −267.93 + 0.68 × age (years) +0.03 × BMI (kg/m^2^) +4.00 × WC (cm) + 22.00 × log10 [TG (mmol/L)] −16.32 × HDL-C (mmol/L); and for females, CVAI= −187.32 + 1.71 × age (years) + 4.32 × BMI (kg/m^2^) + 1.12 × WC (cm) + 39.76 × log10 [TG (mmol/L)] − 11.66 × HDL-C (mmol/L) (22).

### 2.4 Definition of CMM

We collected participants’ medical histories using a questionnaire, and these histories were confirmed based on self-reports of physician diagnoses. CMM is commonly defined as the presence of two or more concurrent cardiometabolic diseases. For this study, we defined CMM as the occurrence of at least one of the following cardiometabolic diseases in conjunction with hypertension: diabetes, coronary heart disease, or stroke (1, 28).

### 2.5 Statistical analysis

Participants were categorized according to their CMM status. The normality of continuous variables was assessed using the Kolmogorov–Smirnov test. Normally distributed continuous variables were presented as mean and standard deviation (SD), while skewed continuous variables were described using the median and interquartile range (IQR). Categorical variables were presented as frequencies and percentages. Differences across the various CMM status groups were assessed using the chi-square test for categorical variables and either t-test or Wilcoxon rank-sum test for continuous variables. Outliers were excluded if they fell below the first quartile (Q1-1.5) or above the third quartile (Q3 + 1.5) (29, 30). **Supplementary Table S1** provides a comparison of the characteristics between the excluded participants and those retained in the study.

We explored the association of CMI, LAP, VAI, and CVAI as continuous variables and categorized into quartiles with the CMM. Multivariable logistic regression models were applied to evaluate the impact of CMI, LAP, VAI, and CVAI on CMM risk, and the associations were expressed as odds ratios (ORs) and 95% confidence intervals (CIs). Potential confounders were selected based on a comparison of factors between the non-CMM and CMM groups, taking into consideration previous relevant studies. The following potential confounding variables were included in the multivariable model: age, sex, ethnicity, education level, marital status, BMI, dietary habits, physical activity, smoking, alcohol consumption, SBP, and DBP. Additionally, we utilized restricted cubic spline (RCS) regression based on multivariable adjusted logistics regression to examine the potential nonlinear relationship. Receiver operating characteristic (ROC) curves were used to calculate the area under the ROC curve (AUC) to assess and compare the discriminatory performance of each adiposity indicators, including CMI, LAP, VAI, and CVAI, in identifying the risk of CMM.

Stratified analysis was conducted by age group (<65 or ≥65 years), sex (males or females), BMI (<24 or ≥24 kg/m^2^), dietary habits (unhealthy or healthy), physical activity (non-active or active), smoking (yes or no), alcohol consumption (yes or no), SBP (<140 or ≥140 mmHg), and DBP (<90 or ≥90 mmHg). To assess potential interactive effects, interaction terms were included in the multiple regression model. Sensitivity analyses were conducted to assess robustness of our findings. We examined the association between adiposity indicators and the risk of CMM without excluding outliers. Furthermore, to account for potential residual confounding factors, we additionally adjusted for TC and LDL-C in the analytical models.

All analyses were performed using R software (4.3.0). A two-sided *P*-value less than 0.05 was considered significant.

## 3 Results

### 3.1 Characteristics of participants

A total of 229,287 participants with hypertension were included in the present study, of which 220,020 (95.96%) participants were classified into the non-CMM group and 9267 (4.04%) into the CMM group. The distribution of CMM is shown in **Supplementary Table S2**. Among all participants, the median (IQR) age was 69.41 (10.65) years, with 92,126 (40.18%) being male and 137,161 (59.82%) being female. Comparing the non-CMM group to the CMM group, it was observed that more participants in the former were younger, female, had a higher level of education, were married, exhibited healthy dietary habits, engaged in less physical activity, were non-smokers but drinkers, had higher SBP, DBP, TC, LDL-C, and HDL-C levels, and had lower BMI, CMI, LAP, VAI, and CVAI levels. There were no significant differences in terms of ethnicity, and TG level (*P >* 0.05) (**Table 1**).

**Table 1:** Basic characteristics according to CMM status.

Characteristic	Total (n=229,287)	Non-CMM (n=220,020)	CMM (n=9267)	*P* value
Age (years), median (IQR)	69.41 (10.65)	69.25 (10.62)	72.85 (11.26)	<0.001 ^a^
BMI (kg/m^2^), median (IQR)	24.50 (4.32)	24.49 (4.32)	24.67 (4.28)	<0.001 ^a^
Sex, n (%)				<0.001 ^b^
Male	92,126 (40.18)	88,057 (40.02)	4069 (43.91)	
Female	137,161 (59.82)	131,963 (59.98)	5198 (56.09)	
Ethnicity, n (%)				0.554 ^b^
Han	228,933 (99.85)	219,683 (99.85)	9250 (99.82)	
Others	354 (0.15)	337 (0.15)	17 (0.18)	
Education level, n (%)				<0.001 ^b^
Primary school or below	66,234 (28.89)	63,398 (28.81)	2836 (30.60)	
Junior high school	39,878 (17.39)	38,124 (17.33)	1754 (18.93)	
Senior high school/Secondary technical school	46,013 (20.07)	43,917 (19.96)	2096 (22.62)	
College or above	77,162 (33.65)	74,581 (33.90)	2581 (27.85)	
Married	204,824 (89.33)	196,882 (89.48)	7942 (85.70)	<0.001 ^b^
Healthy dietary habits, n (%)	220,082 (95.99)	211,260 (96.02)	8822 (95.20)	<0.001 ^b^
Active physical activity, n (%)	129,839 (56.63)	124,430 (56.55)	5409 (58.37)	0.001 ^b^
Smoking, n (%)	36,276 (15.82)	34,716 (15.78)	1560 (16.83)	0.007 ^b^
Alcohol drinking, n (%)	21,608 (9.42)	20,799 (9.45)	809 (8.73)	0.021 ^b^
SBP (mmHg), median (IQR)	137.00 (19.50)	137.00 (19.50)	135.50 (19.00)	<0.001 ^a^
DBP (mmHg), median (IQR)	80.00 (12.00)	80.00 (12.00)	77.00 (11.50)	<0.001 ^a^
TC (mmol/L), median (IQR)	5.14 (1.50)	5.16 (1.50)	4.48 (1.63)	<0.001 ^a^
TG (mmol/L), median (IQR)	1.44 (0.95)	1.44 (0.95)	1.44 (0.95)	0.813 ^a^
LDL-C (mmol/L), median (IQR)	3.07 (1.28)	3.09 (1.27)	2.55 (1.34)	<0.001 ^a^
HDL-C (mmol/L), median (IQR)	1.35 (0.45)	1.35 (0.45)	1.26 (0.41)	<0.001 ^a^
CMI, median (IQR)	0.59 (0.53)	0.59 (0.52)	0.64 (0.55)	<0.001 ^a^
LAP, median (IQR)	35.96 (32.21)	35.91 (32.24)	37.20 (32.55)	<0.001 ^a^
VAI, median (IQR)	1.77 (1.57)	1.77 (1.56)	1.89 (1.66)	<0.001 ^a^
CVAI, median (IQR)	118.63 (43.05)	118.25 (43.03)	128.25 (42.25)	<0.001 ^a^

CMM, cardiometabolic multimorbidity; IQR, interquartile range; BMI, body mass index; SBP, systolic blood pressure; DBP, diastolic blood pressure; TC, total cholesterol; TG, triglyceride; LDL-C, low-density lipoprotein cholesterol; HDL-C, high-density lipoprotein cholesterol; CMI, cardiometabolic index; LAP, lipid accumulation product; VAI, visceral adiposity index; CVAI, Chinese visceral adiposity index.

a P value from Wilcoxon rank sum test.

b P value Chi-square test.

### 3.2 Adiposity indicators and CMM

**Figure 2:**
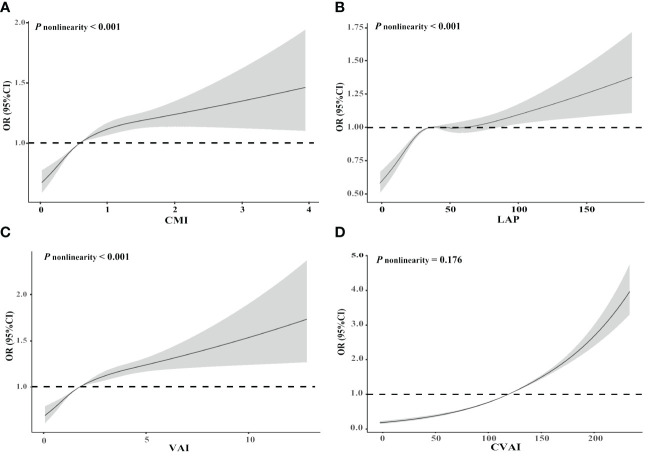
Restricted cubic spline of the relationship of cardiometabolic multimorbidity with CMI **(A)**, LAP **(B)**, VAI **(C)**, and CVAI **(D)**. Potential nonlinear relationships were examined using restricted cubic splines, with odd ratios (ORs) based on logistic regression models. The ORs was adjusted for age, sex, ethnicity, education level, marital status, body mass index, dietary habits, physical activity, smoking, alcohol drinking, systolic blood pressure, and diastolic blood pressure. CMI, cardiometabolic index; LAP, lipid accumulation product; VAI, visceral adiposity index; CVAI, Chinese visceral adiposity index.

The risk of CMM exhibited a positive association with increasing levels of CMI, LAP, VAI, and CVAI (**Table 2**). After considering possible confounders, each 1- standard deviation increase in CMI, LAP, VAI, and CVAI was associated with a 14% (OR 1.14, 95% CI 1.12, 1.17), 8% (OR 1.08, 95% CI 1.06, 1.11), 12% (OR 1.12, 95% CI 1.10, 1.15), and 54% (OR 1.54, 95% CI 1.49, 1.59) increased risk of CMM, respectively. When comparing individuals in the highest quartile of CMI, LAP, VAI, and CVAI with those in the lowest quartile, the former group had a 1.39-fold (95% CI 1.30, 1.48, *P*_trend_ < 0.001), 1.28-fold (95% CI 1.19, 1.37, *P*_trend_ < 0.001), 1.37-fold (95% CI 1.29, 1.46, *P*_trend_ < 0.001), and 2.56-fold (95% CI 2.34, 2.79, *P*_trend_ < 0.001) increased risk of CMM after adjusting for potential confounders. **Figure 2** illustrates the associations of CMI, LAP, and VAI with CMM risk using RCS curves with four knots, revealing significant nonlinear associations (all *P* nonlinearity < 0.001). Nevertheless, no nonlinear association was observed between CVAI and CMM (*P* nonlinearity = 0.176). CMM risk exhibited a rapid increase with rising levels of CMI, LAP, and VAI until approximately 1, 40, and 5, respectively, after which the increment slowed down.

**Table 2:** Association between adiposity indicators and CMM in logistic regression models.

	No	Unadjusted model		Adjusted model	
		OR (95% CI)	*P*	OR (95% CI)	*P*
CMI					
Per SD change		1.12 (1.10, 1.14)	<0.001	1.14 (1.12, 1.17)	<0.001
Quartiles of CMI					
Q1 (≤0.38)	57,322	1.00 (reference)		1.00 (reference)	
Q2 (0.39-0.59)	57,324	1.14 (1.07, 1.22)	<0.001	1.12 (1.05, 1.19)	<0.001
Q3 (0.60-0.91)	57,319	1.26 (1.19, 1.34)	<0.001	1.24 (1.17, 1.32)	<0.001
Q4 (>0.91)	57,322	1.40 (1.32, 1.49)	<0.001	1.39 (1.30, 1.48)	<0.001
*P*_trend_			<0.001		<0.001
LAP					
Per SD change		1.06 (1.04, 1.08)	0.002	1.08 (1.06, 1.11)	<0.001
Quartiles of LAP					
Q1 (≤22.60)	57,371	1.00 (reference)		1.00 (reference)	
Q2 (22.61-35.96)	57,284	1.17 (1.10, 1.24)	<0.001	1.18 (1.11, 1.26)	<0.001
Q3 (35.97-54.81)	57,378	1.16 (1.09, 1.23)	<0.001	1.19 (1.11, 1.27)	<0.001
Q4 (>54.81)	57,254	1.21 (1.14, 1.29)	<0.001	1.28 (1.19, 1.37)	<0.001
*P*_trend_			<0.001		<0.001
VAI					
Per SD change		1.09 (1.07, 1.12)	<0.001	1.12 (1.10, 1.15)	<0.001
Quartiles of VAI					
Q1 (≤1.15)	57,322	1.00 (reference)		1.00 (reference)	
Q2 (1.16-1.77)	57,321	1.08 (1.01, 1.15)	0.016	1.11 (1.04, 1.18)	0.001
Q3 (1.78-2.72)	57,322	1.19 (1.12, 1.27)	<0.001	1.25 (1.18, 1.33)	<0.001
Q4 (>2.72)	57,322	1.26 (1.19, 1.34)	<0.001	1.37 (1.29, 1.46)	<0.001
*P*_trend_			<0.001		<0.001
CVAI					
Per SD change		1.37 (1.34, 1.40)		1.54 (1.49, 1.59)	
Quartiles of CVAI					
Q1 (≤96.95)	57,322	1.00 (reference)		1.00 (reference)	
Q2 (96.96-118.63)	57,321	1.43 (1.33, 1.53)	<0.001	1.49 (1.39, 1.60)	<0.001
Q3 (118.64-140.00)	57,322	1.71 (1.60, 1.82)	<0.001	1.82 (1.69, 1.96)	<0.001
Q4 (>140.00)	57,322	2.29 (2.15, 2.43)	<0.001	2.56 (2.34, 2.79)	<0.001
*P*_trend_			<0.001		<0.001

OR, odds ratio; CI, confidence interval; CMM, cardiometabolic multimorbidity; CMI, cardiometabolic index; SD, standard deviation; LAP, lipid accumulation product; VAI, visceral adiposity index; CVAI, Chinese visceral adiposity index. Adjustment for age, sex, ethnicity, education level, marital status, body mass index, dietary habits, physical activity, smoking, alcohol drinking, systolic blood pressure, and diastolic blood pressure.

### 3.3 Identification ability of adiposity indicators for CMM

**Figure 3:**
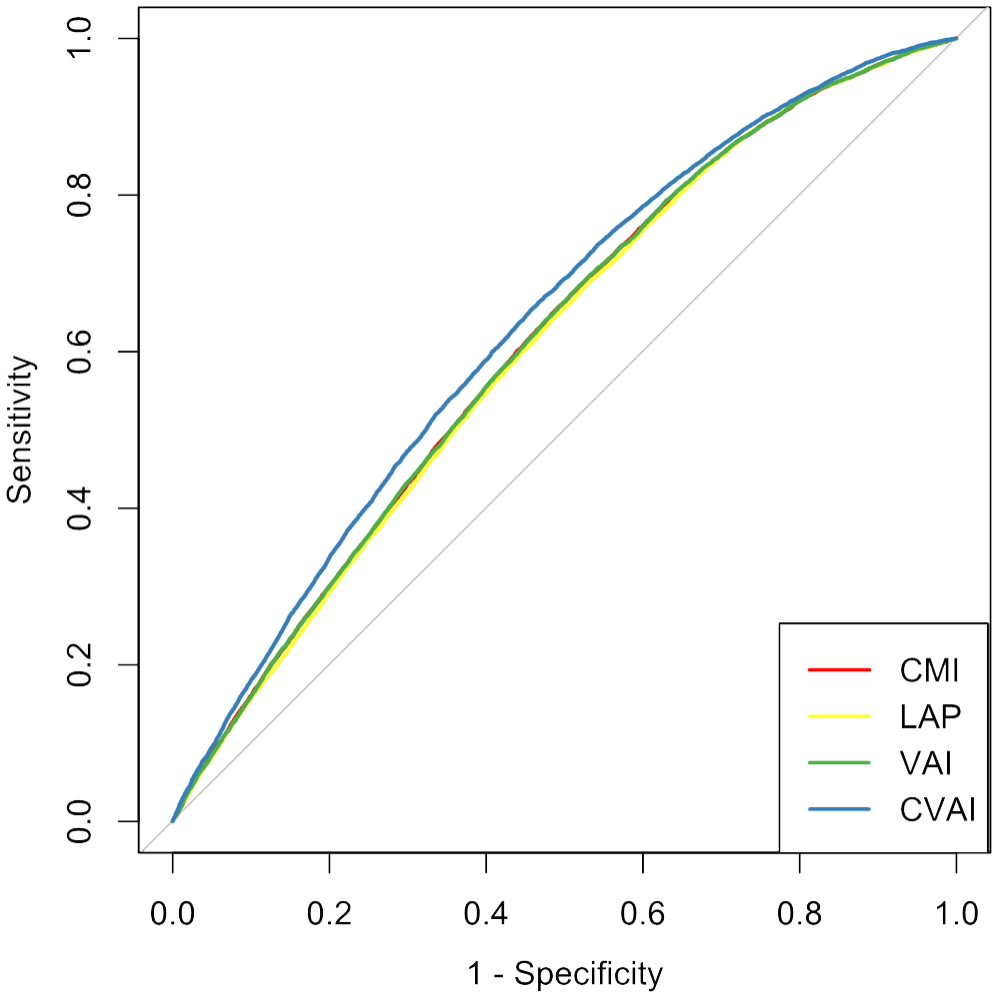
Receiver operating characteristic curves for adiposity indicators for predicting cardiometabolic multimorbidity among hypertension participants. CMI, cardiometabolic index; LAP, lipid accumulation product; VAI, visceral adiposity index; CVAI, Chinese visceral adiposity index.

The ROC curves and corresponding AUCs for CMI, LAP, VAI, and CVAI in identifying CMM are shown in **Table 3** and **Figure 3**. The adjusted AUCs (95% CIs) were 0.612 (0.607, 0.618), 0.608 (0.602, 0.613), 0.612 (0.607, 0.618), and 0.634 (0.628, 0.639), respectively. Notably, CVAI exhibited the highest AUC among all the adiposity indices included in this analysis.

**Table 3:** Predictive performance of adiposity indicators for cardiometabolic multimorbidity in hypertension patients.

Indicators	AUC (95% CI)	Adjusted AUC (95% CI)	*P* for comparison
CVAI	0.588(0.583, 0.594)	0.634 (0.628, 0.639)	Reference
CMI	0.538 (0.532, 0.544)	0.612 (0.607, 0.618)	< 0.001
LAP	0.520 (0.514, 0.526)	0.608 (0.602, 0.613)	< 0.001
VAI	0.529 (0.523, 0.535)	0.612 (0.607, 0.618)	< 0.001

CI, confidence interval; CMI, cardiometabolic index; LAP, lipid accumulation product; VAI, visceral adiposity index; CVAI, Chinese visceral adiposity index. Adjustment for age, sex, ethnicity, education level, marital status, body mass index, dietary habits, physical activity, smoking, and alcohol drinking.

### 3.4 Stratified analysis

In the stratified analysis, the positive associations of CMI, LAP, VAI, and CVAI with CMM risk remained statistically significant in all subgroups, except for the dietary habits and alcohol drinking subgroup (**Table 4**). Notably, the association between CMI and CMM risk appeared to be more pronounced in individuals with a BMI < 24 kg/m^2^ (*P*_interaction_ < 0.05). Similarly, the association between LAP and CMM risk was stronger in subjects with BMI < 24 kg/m^2^ and SBP ≥ 140mmHg (both *P*_interaction_ < 0.05). Additionally, the association between VAI and CMM risk exhibited greater significance in individuals aged ≥ 65 years and those with a BMI < 24 kg/m^2^ (all *P*_interaction_ < 0.05). The association between CVAI and CMM risk appeared to be more pronounced in females, individuals with unhealthy dietary habits, and those were non-active physical activity (all *P*_interaction_ < 0.05). No significant interactions were found between other factors and CMI, LAP, VAI, or CVAI.

**Table 4:** Stratified analyses for the association between adiposity indicators and cardiometabolic multimorbidity stratified by different factors.

Characteristics	No		CMI		LAP		VAI		CVAI	
	Non-CMM	CMM	OR (95%CI)	*P*_interaction_	OR (95%CI)	*P*_interaction_	OR (95%CI)	*P*_interaction_	OR (95%CI)	*P*_interaction_
Age, years				0.259		0.285		0.035		0.398
<65	59,060	1151	1.08 (1.02, 1.14)		1.10 (1.03, 1.17)		1.10 (1.03, 1.16)		1.78 (1.61, 1.96)	
≥65	160,960	8116	1.12 (1.09, 1.14)		1.08 (1.05, 1.10)		1.13 (1.10, 1.15)		1.50 (1.45, 1.55)	
Sex				0.578		0.090		0.306		<0.001
Males	88,057	4069	1.09 (1.06, 1.12)		1.07 (1.03, 1.11)		1.13 (1.08, 1.17)		1.40 (1.34, 1.47)	
Females	131,963	5198	1.13 (1.10, 1.16)		1.08 (1.05, 1.12)		1.12 (1.09, 1.15)		1.80 (1.70, 1.90)	
BMI, kg/m^2^				<0.001		<0.001		<0.001		0.956
<24	96,441	3857	1.20 (1.16, 1.24)		1.20 (1.15, 1.25)		1.19 (1.15, 1.23)		1.48 (1.41, 1.54)	
≥24	123,579	5410	1.09 (1.06, 1.11)		1.07 (1.04, 1.10)		1.09 (1.06, 1.12)		1.38 (1.33, 1.42)	
Dietary habits				0.772		0.468		0.865		0.042
Unhealthy	8760	445	1.10 (1.01, 1.21)		1.08 (0.97, 1.21)		1.13 (1.03, 1.24)		1.69 (1.44, 1.98)	
Healthy	211,260	8822	1.12 (1.09, 1.14)		1.08 (1.05, 1.11)		1.12 (1.10, 1.15)		1.53 (1.48, 1.58)	
Physical activity				0.280		0.133		0.064		0.003
Non-active	95,590	3858	1.14 (1.10, 1.17)		1.11 (1.07, 1.15)		1.14 (1.11, 1.18)		1.64 (1.56, 1.73)	
Active	124,430	5409	1.10 (1.07, 1.13)		1.06 (1.03, 1.09)		1.11 (1.08, 1.14)		1.45 (1.39, 1.52)	
Smoking				0.913		0.054		0.222		0.173
Yes	34,716	1560	1.09 (1.04, 1.14)		1.06 (1.00, 1.13)		1.12 (1.10, 1.15)		1.43 (1.33, 1.54)	
No	185,304	7707	1.12 (1.09, 1.14)		1.08 (1.05, 1.11)		1.12 (1.05, 1.18)		1.56 (1.50, 1.62)	
Alcohol drinking				0.747		0.537		0.719		0.446
Yes	20,799	809	1.09 (1.02, 1.17)		1.08 (0.99, 1.17)		1.11 (1.02, 1.21)		1.55 (1.40, 1.71)	
No	199,221	8458	1.12 (1.09, 1.14)		1.08 (1.05, 1.11)		1.12 (1.10, 1.15)		1.54 (1.49, 1.60)	
SBP, mmHg				0.121		0.002		0.125		0.136
<140	123,325	5701	1.11 (1.08, 1.14)		1.05 (1.02, 1.09)		1.11 (1.08, 1.14)		1.48 (1.41, 1.54)	
≥140	96,695	3566	1.13 (1.09, 1.17)		1.13 (1.08, 1.17)		1.14 (1.10, 1.18)		1.64 (1.56, 1.73)	
DBP, mmHg				0.799		0.155		0.541		0.256
<90	188,285	8537	1.11 (1.09, 1.14)		1.07 (1.04, 1.10)		1.12 (1.10, 1.14)		1.57 (1.51, 1.62)	
≥90	31,735	730	1.10 (1.03, 1.18)		1.14 (1.05, 1.23)		1.12 (1.04, 1.20)		1.76 (1.56, 1.97)	

OR, odd ratio; CI, confidence interval; CMM, cardiometabolic multimorbidity; BMI, body mass index; SBP, systolic blood pressure; DBP, diastolic blood pressure; CMI, cardiometabolic index; LAP, lipid accumulation product; VAI, visceral adiposity index; CVAI, Chinese visceral adiposity index. Adjustment for age, sex, ethnicity, education level, marital status, body mass index, dietary habits, physical activity, smoking, alcohol drinking, systolic blood pressure, and diastolic blood pressure.

### 3.5 Sensitivity analyses

In the sensitivity analysis, we observed consistent findings regarding the association between adiposity indicators and the risk of CMM when outliers were not excluded (**Supplementary Table S3**). Furthermore, additional adjustment for TC and LDL-C had minimal impact on the results (**Supplementary Table S4**).

## 4 Discussion

This is the first study to investigate the association of CMI, LAP, VAI, and CVAI with the risk of CMM in Chinese hypertensive patients. Our findings demonstrated that CMI, LAP, VAI, and CVAI were positively and independently associated with CMM among individuals with hypertension, even after adjusting for other confounding factors. Additionally, it was observed that the levels of CMI, LAP, and VAI displayed a nonlinear relationship with CMM. Furthermore, CVAI demonstrated the highest predictive for CMM compared to CMI, LAP, and VAI.

CMI offers the advantage of applicability in assessing various conditions such as type 2 diabetes mellitus, stroke, and atherosclerotic progression (11, 31, 32). It has been found to be associated with an increased risk of diabetes in both Chinese and Japanese adult populations (11, 33). A large cross-sectional study conducted in rural China showed that for each 1 standard deviation increment in CMI, the risk of ischemic stroke increased by 18% for females and 14% for males (31). Moreover, Wakabayashi et al. reported a correlation between CMI and the severity of atherosclerosis in individuals with peripheral arterial disease (32). However, no prior studies have explored the impact of CMI on CMM risk. The present study was initiated to examine the utility of CMI in identifying individuals with CMM among hypertensive patients. Our findings strongly suggest that higher CMI values are closely associated with CMM.

LAP has emerged as a promising anthropometric index for assessing cardiometabolic risk (31, 34). Evidence showed that LAP serves as a predictor of diabetes risk in both Japanese and Chinese populations, outperforming traditional obesity indicators (35, 36). A population-based prospective cohort study conducted in China revealed that individuals in the high LAP group had a 1.67 times higher risk of stroke compared to those in the low LAP group (37). Furthermore, LAP has been reported to exhibit a positive association with the incidence of cardiovascular events and all-cause mortality (38, 39). In line with these findings, the present study identified a positive association between LAP and the risk of CMM in hypertensive patients.

VAI has been linked to the development of various cardiometabolic diseases, atherosclerosis, and stroke (12, 16, 40). A meta-analysis, including 9 cohort studies, found that for each 1-unit increase in VAI, there was a 42% higher risk of type 2 diabetes mellitus (41). The National Health and Nutrition Examination Survey, including 29,337 adults, showed that VAI was associated with a 12% increase in the prevalence of stroke (16). Han et al. revealed a strong association between VAI and the severity of coronary heart disease in Chinese adults (42). Furthermore, findings from the UK Biobank cohort study showed a positive relationship between VAI and the increased risk of all-cause mortality as well as cause-specific mortalities (43). In alignment with these findings, our study revealed that each 1- standard deviation increase in VAI was associated with a 12% increased risk of CMM.

CVAI is considered to be a reliable indicator of visceral adipose scores specific to the Chinese population, and it has been used to assess various diseases, including diabetes, stroke, and cardiovascular events (22, 44, 45). Studies have shown that CVAI is associated with an increased risk of diabetes in both Chinese and Japanese adult populations (22, 46). The China Health and Retirement Longitudinal Study, including 7242 middle-aged and elderly residents, showed that CVAI was associated with a 17% increased risk of incident stroke (44). Two cohort studies conducted in Xinjiang and Tangshan showed that CVAI was associated with an elevated risk of cardiovascular events in patients with hypertension and type 2 diabetes (45, 47). In line with these findings, the present study identified a positive association between CVAI and the risk of CMM in hypertensive patients. Furthermore, we discovered that CVAI exhibited the highest predictive capacity for CMM compared to CMI, LAP, and VAI. In line with our results, the Rural Chinese Cohort Study found that CVAI was the most accurate predictor of stroke (20). Additionally, a cross-sectional study including 2514 Chinese women aged between 45 and 55 years showed that CVAI outperformed five other obesity indicators as a predictor of metabolic dysfunction-associated fatty liver disease (48). Moreover, data from the China Nutrition and Health Surveillance showed that CVAI was the most robust predictor of hypertension in both men and women, surpassing other obesity indices (49). These findings suggest that CVAI may serve as a valuable and easily measurable indicator for identifying individuals at high risk of CMM.

The RCS analysis suggested nonlinear associations of CMI, LAP, and VAI with the risk of CMM. At the initial stage of increasing CMI, LAP, and VAI, the risk of CMM increased rapidly, but the rate of increase subsequently slowed down. A cross-sectional study conducted in Japan demonstrated a nonlinear dose-response association between CMI and the risk of non-alcoholic fatty liver disease (50). Furthermore, RCS results from a community-based prospective cohort in Lishui, China observed that elevated LAP level was associated with a higher atherosclerotic burden in coronary arteries (51). Similarly, the National Health and Nutrition Examination Survey identified a significant nonlinear association between VAI and all-cause mortality (43). However, our finding did not observe a nonlinear association between CVAI and CMM. Similarly, a cohort study found no nonlinear relationship between CVAI and the risk of diabetes mellitus in non-obese Japanese adults (52). Additionally, the China Health and Retirement Longitudinal Study found no evidence of a nonlinear link between CVAI and the incidence of stroke (44). Overall, the findings from various dose-response analyses across different studies have consistently indicated that higher levels of CMI, LAP, and VAI are linked to an increased diseases burden and mortality, including hypertension. We believe that further investigation into the mechanisms underlying this nonlinear dose-response relationship between visceral obesity and CMM is warranted in future research.

Although the exact mechanisms underlying the association between these adiposity indicators and CMM among hypertensive patients are unclear, several hypotheses have been proposed. First, obesity may lead to elevated levels of inflammatory cytokines such as tumor necrosis factor-α, interleukin-6, and C-reactive protein (53). These inflammatory cytokines play a crucial role in the development and progression of metabolic regulation disorders and atherosclerotic plaques on blood vessel walls, which could ultimately contribute to CMM (54, 55). Second, previous evidence has demonstrated that obesity can lead to endothelial dysfunction, further contributing to insulin resistance (56, 57). Insulin resistance results in increased basal lipolysis in adipose tissue, the releasing free fatty acids into circulation (58). Excess free fatty acids are generated outside of fat storage tissue and transported to ectopic locations such as the viscera organs, the heart, and vasculature, eventually contributing to the development of CMM (59). Third, abdominal fat has been associated with elevated cortisol levels due to the possible hyperactivity of the hypothalamic-pituitary-adrenocortical axis and local adipocyte cortisol production (60). Long-term exposure to high level of cortisol may disrupt glucose homeostasis, induce insulin resistance, elevate blood pressure, and increase triglyceride levels, ultimately leading to the development of CMM (61, 62). Finally, individuals with obesity often adopt unhealthy lifestyles, including physical inactivity, excessive calorie intake, smoking, and excessive alcohol consumption, all of which are recognized risk factors for CMM (63, 64). Our study demonstrated a positive association of CMI, LAP, VAI, and CVAI with CMM, suggesting that these adiposity indicators could potentially serve as simple and effective markers for assessing CMM risk. However, further mechanistic studies are needed to elucidate the specific roles of these indicators in the development of CMM among hypertensive patients.

This study possesses several strengths that deserve recognition. Firstly, it is the first investigation to examine the associations of adiposity indicators, including CMI, LAP, VAI, and CVAI, with the risk of CMM, especially among a substantial large group of hypertensive individuals. Secondly, utilizing a large sample of Chinese hypertensive adults, uncommon in similar investigations, our study enhances the statistical power and validity. Additionally, the analytical approach, treating CMI, LAP, VAI, and CVAI as both continuous and categorical variables, alongside conducting sensitivity analysis and trend tests, enhances the robustness and reliability of our results. Furthermore, the consistency of our primary findings across subgroups underscores the stability of our results. However, there are also several limitations to consider in this study. Firstly, a large proportion of participants were excluded due to outliers, potentially impacting the generalizability of our findings. Nevertheless, when including outliers in the analysis, we obtained results consistent with the main findings, reinforcing the robustness of our conclusions. Secondly, the identification of CMM relied on self-reported information obtained through interviews, a common procedure in substantial epidemiological investigations (65, 66). This method may introduce recall bias, potentially leading to an underestimation of CMM prevalence. Nevertheless, the significant associations found between the CMI, LAP, VAI, CVAI, and CMM emphasize the importance of our observations, despite the acknowledged limitations. Thirdly, the definition of a healthy dietary habits relied solely on self-reported data. Therefore, our definition of a healthy dietary habits was limited to our study population and there may be restrictions to extrapolation of results. Fourthly, the cross-sectional nature of this study limits our ability to establish causal relationships between abdominal obesity indices and CMM in hypertensive individuals. However, it serves as a preliminary step towards further longitudinal studies. Consequently, there is a pressing need for future longitudinal studies to further investigate the role of CMI, LAP, VAI, and CVAI in the development of CMM. Fifthly, despite our efforts to adjust for potential risk factors in multivariable analyses, there remains the possibility of residual or unassessed confounding variables influencing our results. Lastly, acknowledging the diverse factors, including economic, cultural, dietary differences and genetic diversity, it needs for caution when extrapolating our data and results to other populations.

## 5 Conclusions

This study is the first to investigate the association of CMI, LAP, VAI, and CVAI with the risk of CMM among Chinese hypertensive patients. This cross-sectional study has revealed significant association of CMI, LAP, VAI, and CVAI with CMM in Chinese adults diagnosed with hypertension. Notably, among these obesity indicators, CVAI demonstrated superior performance in predicting CMM risk. The findings from this study suggest that CVAI might serve as a valuable tool for identifying the risk of CMM in Chinese hypertensive patients. The global prevalence of CMM remains high, underlining the importance of bolstering disease management for hypertensive patients through lifestyle interventions and effective treatment aimed at preventing visceral obesity and, consequently, reducing the incidence of CMM. Additionally, the utility and validity of CMI, LAP, VAI, and CVAI as reliable indicators for the prevention of new-onset CMM warrant further investigation and exploration.

## Data availability statement

The raw data supporting the conclusions of this article will be made available by the authors, without undue reservation.

## Ethics statement

The studies involving humans were approved by the ethical committee of the School of Public Health of Sun Yat-Sen University. The studies were conducted in accordance with the local legislation and institutional requirements. The ethics committee/institutional review board waived the requirement of written informed consent for participation from the participants or the participants’ legal guardians/next of kin because Informed consent did not apply due to the use of secondary data.

## Author contributions

TD: Conceptualization, Formal analysis, Writing – original draft. WL: Conceptualization, Data curation, Formal analysis, Funding acquisition, Writing – original draft. QZ: Data curation, Funding acquisition, Writing – original draft. YY: Data curation, Writing – original draft. XL: Data curation, Writing – original draft. JC: Data curation, Writing – original draft. HL: Conceptualization, Data curation, Funding acquisition, Writing – review & editing. CZ: Conceptualization, Formal analysis, Writing – review & editing.
