# Supplementary material for: Association of adiposity indicators with cardiometabolic multimorbidity risk in hypertensive patients: a large cross-sectional study
Source: Front Endocrinol (Lausanne). 2024 Mar 21;15:1302296. doi: 10.3389/fendo.2024.1302296 (PMC10991765; doi:10.3389/fendo.2024.1302296)
Supplement: Supplementary file 1 [file Table_1.docx]

**Table S1** Characteristics for the deleted participants and retained participants

| Characteristics | Deleted participants  (N= 59,728) | Retained participants (N= 229,287) | *P* value |
| --- | --- | --- | --- |
| Age, years,  median (IQR) | 66.43 (13.76) | 69.41 (10.65) | <0.001 ^a^ |
| Sex, n (%) |  |  | <0.001 ^b^ |
| Male | 23,443 (39.25) | 92,126 (40.18) |  |
| Female | 36,285 (60.75) | 137,161 (59.82) |  |
| Marital status, n (%) |  |  | 0.001 ^b^ |
| Married | 53,638 (89.80) | 204,824 (89.33) |  |
| Others | 6090 (10.20) | 24,463 (10.67) |  |

IQR, interquartile range.

^a^ *P* value from Wilcoxon rank sum test.

^b^ *P* value Chi-square test.
