# Supplementary material for: Association of adiposity indicators with cardiometabolic multimorbidity risk in hypertensive patients: a large cross-sectional study
Source: Front Endocrinol (Lausanne). 2024 Mar 21;15:1302296. doi: 10.3389/fendo.2024.1302296 (PMC10991765; doi:10.3389/fendo.2024.1302296)
Supplement: Supplementary file 2 [file Table_2.docx]

**Table S2** Distribution of CMM status

|  | No | % |
| --- | --- | --- |
| Single CMM |  |  |
| CHD | 21,738 | 9.48 |
| Stroke | 6197 | 2.70 |
| Diabetes | 57,314 | 25.00 |
| CMM |  |  |
| CHD and stroke | 700 | 0.31 |
| CHD and diabetes | 6697 | 2.92 |
| Stroke and diabetes | 1543 | 0.67 |
| CHD and stroke and diabetes | 327 | 0.14 |

CMM, cardiometabolic multimorbidity; CHD, coronary heart disease.
