# Supplementary material for: Association of adiposity indicators with cardiometabolic multimorbidity risk in hypertensive patients: a large cross-sectional study
Source: Front Endocrinol (Lausanne). 2024 Mar 21;15:1302296. doi: 10.3389/fendo.2024.1302296 (PMC10991765; doi:10.3389/fendo.2024.1302296)
Supplement: Supplementary file 3 [file Table_3.docx]

**Table S3** Association between adiposity indicators and the risk of cardiometabolic multimorbidity without deleting outliers (N=273,537)

|  | Unadjusted model | | Adjusted model | |
| --- | --- | --- | --- | --- |
|  | OR (95% CI) | *P* | OR (95% CI) | *P* |
| *CMI* |  |  |  |  |
| Per SD change | 1.02 (1.01, 1.03) | <0.001 | 1.03 (1.02, 1.04) | <0.001 |
| Quartiles of CMI |  |  |  |  |
| Q1 (≤0.39) | 1.00 (reference) |  | 1.00 (reference) |  |
| Q2 (0.40-0.62) | 1.18 (1.11, 1.25) | <0.001 | 1.21 (1.14, 1.28) | <0.001 |
| Q3 (0.63-1.00) | 1.31 (1.24, 1.39) | <0.001 | 1.38 (1.30, 1.46) | <0.001 |
| Q4 (>1.00) | 1.40 (1.32, 1.48) | <0.001 | 1.55 (1.47, 1.64) | <0.001 |
| *P* value for trend |  | <0.001 |  | <0.001 |
| *LAP* |  |  |  |  |
| Per SD change | 1.02 (1.01, 1.03) | 0.002 | 1.03 (1.02, 1.04) | <0.001 |
| Quartiles of LAP |  |  |  |  |
| Q1 (≤23.20) | 1.00 (reference) |  | 1.00 (reference) |  |
| Q2 (23.21-38.00) | 1.18 (1.11, 1.24) | <0.001 | 1.26 (1.20, 1.34) | <0.001 |
| Q3 (38.01-60.84) | 1.18 (1.12, 1.25) | <0.001 | 1.33 (1.25, 1.41) | <0.001 |
| Q4 (>60.84) | 1.24 (1.17, 1.31) | <0.001 | 1.51 (1.43, 1.60) | <0.001 |
| *P* value for trend |  | <0.001 |  | <0.001 |
| *VAI* |  |  |  |  |
| Per SD change | 1.02 (1.01, 1.03) | <0.001 | 1.02 (1.01, 1.03) | <0.001 |
| Quartiles of VAI |  |  |  |  |
| Q1 (≤1.16) | 1.00 (reference) |  | 1.00 (reference) |  |
| Q2 (1.17-1.84) | 1.10 (1.04, 1.17) | <0.001 | 1.17 (1.10, 1.24) | <0.001 |
| Q3 (1.85-2.97) | 1.23 (1.16, 1.30) | <0.001 | 1.36 (1.28, 1.44) | <0.001 |
| Q4 (>2.97) | 1.31 (1.24, 1.38) | <0.001 | 1.53 (1.45, 1.62) | <0.001 |
| *P* value for trend |  | <0.001 |  | <0.001 |
| *CVAI* |  |  |  |  |
| Per SD change | 1.27 (1.22, 1.33) | <0.001 | 1.21 (1.16, 1.26) | <0.001 |
| Quartiles of CVAI |  |  |  |  |
| Q1 (≤97.53) | 1.00 (reference) |  | 1.00 (reference) |  |
| Q2 (97.54-120.24) | 1.44 (1.35, 1.53) | <0.001 | 1.48 (1.38, 1.58) | <0.001 |
| Q3 (120.25-142.72) | 1.74 (1.63, 1.84) | <0.001 | 1.81 (1.69, 1.94) | <0.001 |
| Q4 (>142.73) | 2.30 (2.17, 2.43) | <0.001 | 2.49 (2.30, 2.69) | <0.001 |
| *P* value for trend |  | <0.001 |  | <0.001 |

CMI, cardiometabolic index; LAP, lipid accumulation product; VAI, visceral adiposity index; CVAI, Chinese visceral adiposity index.

Adjustment for age, sex, ethnicity, education level, marital status, body mass index, dietary habits, physical activity, smoking, alcohol drinking, systolic blood pressure, and diastolic blood pressure.
