# Supplementary material for: Association of adiposity indicators with cardiometabolic multimorbidity risk in hypertensive patients: a large cross-sectional study
Source: Front Endocrinol (Lausanne). 2024 Mar 21;15:1302296. doi: 10.3389/fendo.2024.1302296 (PMC10991765; doi:10.3389/fendo.2024.1302296)
Supplement: Supplementary file 4 [file Table_4.docx]

**Table S4** Association between adiposity indicators and the risk of cardiometabolic multimorbidity with additional adjustment for TC and LDL-C

|  | Q1 | Q2 | Q3 | Q4 | *P* _trend_ |
| --- | --- | --- | --- | --- | --- |
| *CMI* |  |  |  |  |  |
| No | 57,322 | 57,324 | 57,319 | 57,322 | - |
| Adjusted OR (95% CI) | 1.00 (reference) | 1.18 (1.10, 1.26) | 1.36 (1.26, 1.46) | 1.54 (1.42, 1.68) | <0.001 |
| *LAP* |  |  |  |  |  |
| No | 57,371 | 57,284 | 57,378 | 57,254 | - |
| Adjusted OR (95% CI) | 1.00 (reference) | 1.30 (1.22, 1.38) | 1.40 (1.31, 1.50) | 1.63 (1.51, 1.76) | <0.001 |
| *VAI* |  |  |  |  |  |
| No | 57,322 | 57,321 | 57,322 | 57,322 | - |
| Adjusted OR (95% CI) | 1.00 (reference) | 1.17 (1.09, 1.25) | 1.37 (1.27, 1.47) | 1.50 (1.38, 1.63) | <0.001 |
| *CVAI* |  |  |  |  |  |
| No | 57,322 | 57,321 | 57,322 | 57,322 | - |
| Adjusted OR (95% CI) | 1.00 (reference) | 1.42 (1.32, 1.52) | 1.68 (1.55, 1.82) | 2.25 (2.06, 2.45) | <0.001 |

TC, total cholesterol; LDL-C, low-density lipoprotein cholesterol; CMI, cardiometabolic index; LAP, lipid accumulation product; VAI, visceral adiposity index; CVAI, Chinese visceral adiposity index.

Adjustment for age, sex, ethnicity, education level, marital status, body mass index, dietary habits, physical activity, smoking, alcohol drinking, systolic blood pressure, diastolic blood pressure, total cholesterol, and low-density lipoprotein cholesterol.
